# Supplementary material for: Etonogestrel-releasing subdermal contraceptive implant: Budget impact analysis based on the Brazilian private healthcare system
Source: PLoS One. 2024 Mar 28;19(3):e0301207. doi: 10.1371/journal.pone.0301207 (PMC10977723; doi:10.1371/journal.pone.0301207)
Supplement: Attachment — Submitted filename: Response to reviewers.docx [file pone.0301207.s002.docx]

**Response to Reviewers**

1. *It is not clear what the study set out to address as the reference to “cost avoidance” did not state who the cost avoidance relates to.*

- Line 38: "... cost avoided by private health system payers."

- Line 371-372: "... could result in a cost avoidance of $7,640,804.02 for payers of the private health system..."

2. *The article appears more like a “marketing strategy” presentation with obvious bias towards the product IMP-ETN and therefore does not qualify for publication in PLOS ONE. This is not surprising judging from the fact that some of the Authors have conflicting interests being employees of the sponsoring organization (ORGANON).*

       Budget impact analyzes are a relevant and increasingly required element by authorities in the health technology reimbursement process. Our work is fully compliant with Good Practice Guideline for Budget Impact Analysis defined by the International Society of Pharmacoeconomics and Outcomes Research – ISPOR (MAUSKOPF et al., 2007; SULLIVAN et al., 2014). Crucially, the Brazilian Government has methodological guidelines in line with ISPOR and which were also fully respected, especially related to the time horizon (BRASIL, 2012).

In the context of the PLOs journal, Babigumira’s et al. (2012) publication presents an economic evaluation that also considers modern methods (implants) for the African region. The inclusion of modern contraceptive methods in the list of private health is the most relevant action to reduce unplanned pregnancies and their harmful effects on health, such as: postpartum depression, unsafe abortions, maternal-fetal mortality, etc. (FILHO et al., 2022).

In Brazil, there is a government publication that ensures cost-effective implants. However, for the private health system whose expenditure corresponds to 55% of the national health budget, there is no publication of results. There is only an abstract publication with results for a single payer which does not reflecting the whole country of Brazil with all the nuances (BUDIB et al., 2023).

References:

Brasil. (2012). Diretrizes metodológicas. Análise de impacto orçamentário. Manual para o Sistema de Saúde do Brasil.

Artigos similares no PLOS: Babigumira, J. B., Stergachis, A., Veenstra, D. L., Gardner, J. S., Ngonzi, J., Mukasa-Kivunike, P., & Garrison, L. P. (2012). Potential cost-effectiveness of universal access to modern contraceptives in Uganda. *PloS one*, *7*(2), e30735.

Mauskopf JA, Sullivan SD, Annemans L, et al. Principles of good practice for budget impact analysis: report of the ISPOR Task Force on Good Research Practices— Budget Impact Analysis. Value Health. 2007;10(5):336-347.

Sullivan SD, Mauskopf JA, Augustovski F, et al. Principles of good practice for budget impact analysis II: report of the ISPOR Task Force on Good Research Practices – Budget Impact Analysis. Value Health. 2014;17(1):5-14.

Brasil. (2021). Implante subdérmico de etonogestrel na prevenção da gravidez não planejada por mulheres adultas em idade reprodutiva entre 18 e 49 anos. Relatório Técnico.

Filho, A. L. D. S., Lino, L. M., Ramires, Y., & Bueno, R. L. (2022). Reproductive health in Brazil and Europe: Commentary on an analytical parallel. *Reproductive, Female and Child Health*, *1*(1), 7-10.

Budib M, Ayache R, Ribeiro N, Appel R Pereira L, Ramires Y, Bueno RL, Lemos E. Usage and Costs Profile of Long-Term Contraception Users at a Health Plan Operator in the Brazil. Value in Health, Volume 26, Issue 6, S2 (June 2023), 2023-05, ISPOR 2023, Boston, MA, USA

4. *As this study derives the outcome figures based on available data, the conclusion is only as good as the data. Therefore more justification on the validity of the data sets, the references, and the figures selected should be added in the methodology section. For example in line 131, it would be easier for readers to understand the methodology by explaining what specific data are retrieved from the ANS, and how comprehensive and accurate are the data on ANS (e.g. any data checks by ANS, any previous study validating data of ANS).*

      Modifications were performed.

5. *It would also be good to explain if the costs defined by CBHPM (line 150-151) and CMED (line 154-155) correlate to the cost actually imposed by the fee-for-service private healthcare sector – as many readers are unfamiliar with the Brazilian medical system, one wonders if the costs in these lists are legally binding or whether private sectors can inflate the price as they wish.*

      Line 288 - 295: " The cost of each type of contraception was calculated based on the use of mandatory Brazilian professional spread sheets, to guarantee comparability throughout the national territory, according to the values defined by the Classificação Brasileira Hierarquizada de Procedimentos Médicos (CBHPM; the Brazilian Hierarchical Classification of Medical Procedures, a referenced tool adopted as a minimum and ethical standard for remuneration of medical procedures for the Supplementary Health) for the insertion and removal of contraceptives with a 4A rating, considering any additional examinations necessary for LNG-IUS insertion [21]."

      Line 295 - 301: The prices of the therapeutic alternatives were retrieved from the 2021 drug price list of the Drug Market Regulation Chamber (Câmara de Regulação do Mercado de Medicamentos – CMED) through the factory price (PF), which is the value to be practiced by the manufacturing, importing, or distributing companies, and the maximum price allowed for sale to pharmacies, drugstores, and public administration entities [22]. These were adjusted by the purchasing power parity (PPP) of Brazil in 2021.

5. *On the other hand, why is ANS data on the actual cost for procedures not used, is it because such data is not available in ANS?*

A ANS tem, porém são dados competitivos o que poderia levar a prática de concorrência desleal entre as empresas. The ANS has, however, these are competitive data, which could lead to the practice of unfair competition between companies.

6. *I am also a bit confused by line 147, which mentions an assumed annual growth of 7% - does this refer to the growth in target population, or growth of IMP-ETN market share? Better clarify that. Also for line 165, is there any justification for the assumed constant growth of 5% per year? I understand it may be difficult to accurately make estimations, and the authors have also acknowledged this issue in the discussion.*

Referenced according to RAC and ANS
