# Supplementary material for: Etonogestrel-releasing subdermal contraceptive implant: Budget impact analysis based on the Brazilian private healthcare system
Source: PLoS One. 2024 Mar 28;19(3):e0301207. doi: 10.1371/journal.pone.0301207 (PMC10977723; doi:10.1371/journal.pone.0301207)
Supplement: Attachment — Submitted filename: Reviewer Comment and Recommendation.docx [file pone.0301207.s001.docx]

Reviewer Comment and Recommendation

This article is an economic analysis that assesses the cost-effectiveness of adopting IMP-ETN over a period of five year compared to LNG-IUS. The author has the strength of presenting evidence in a setting that previous evidence is scarce – few economic analysis has been performed on this topic in the Brazilian setting, and therefore this article would be interesting to readers working in the country and neighbouring region, or even countries that are socio-economically similar. However revisions are needed as charted below.

As this study derives the outcome figures based on available data, the conclusion is only as good as the data. Therefore more justification on the validity of the data sets, the references, and the figures selected should be added in the methodology section. For example in line 131, it would be easier for readers to understand the methodology by explaining what specific data are retrieved from the ANS, and how comprehensive and accurate are the data on ANS (e.g. any data checks by ANS, any previous study validating data of ANS). It would also be good to explain if the costs defined by CBHPM (line 150-151) and CMED (line 154-155) correlate to the cost actually imposed by the fee-for-service private healthcare sector – as many readers are unfamiliar with the Brazilian medical system, one wonders if the costs in these lists are legally binding or whether private sectors can inflate the price as they wish. On the other hand, why is ANS data on the actual cost for procedures not used, is it because such data is not available in ANS?

I am also a bit confused by line 147, which mentions an assumed annual growth of 7% - does this refer to the growth in target population, or growth of IMP-ETN market share? Better clarify that. Also for line 165, is there any justification for the assumed constant growth of 5% per year? I understand it may be difficult to accurately make estimations, and the authors have also acknowledged this issue in the discussion.

Another suggestion I have is to move the literature review (line 186-259) to the introduction or discussion, as the main outcome should be the on the cost-effectiveness analysis.
